# Feline blood donation via cephalic intravenous catheter: A novel method

**DOI:** 10.1002/vetr.5925

**Published:** 2025-12-04

**Authors:** Charlotte Russo, Karen Humm

**Affiliations:** ^1^ Clinical Science and Services Royal Veterinary College Hatfield UK

**Keywords:** blood testing, cats, welfare

## Abstract

**Background:**

Feline jugular blood donation requires heavy sedation or a very compliant cat. Donation using an intravenous catheter requires less restraint and potentially decreases stress.

**Methods:**

This was an observational study of feline blood donors at a veterinary hospital between February and June 2023. Blood donation through a standard cephalic catheter was attempted in cats that were anticipated not to tolerate jugular donation. The percentage haemolysis of the donated packed red blood cells was then calculated for catheter and jugular donations.

**Results:**

Forty‐six donations were performed over the study period. The catheter donation method was used successfully in 13 cats, of which eight were not sedated and five were mildly sedated with butorphanol and/or gabapentin. In four cats, a combination of jugular and catheter donation methods was used (all sedated). In 29 cats, the jugular method alone was used; four of these cats were sedated. The mean percentage haemolysis of the packed red blood cells from 11 catheter donations and 11 jugular donations was 0.21% (standard deviation: 0.11, range: 0.10–0.43%) and 0.20% (standard deviation: 0.14, range: 0.01–0.54%), respectively. These values were not significantly different.

**Limitation:**

Cats had been selected for a calm temperament.

**Conclusion:**

Although not possible in all cats, intravenous catheter donation can be a successful method for blood donation.

## INTRODUCTION

Demand for feline blood products is increasing,^1^ but the requirements of recipients need to be carefully balanced with the welfare of donors. The Royal College of Veterinary Surgeons (RCVS) Code of Professional Conduct currently states that sedation should not be used to enable blood donation, but an exception is made in an emergency, where the donor and recipient animals are known to the veterinary surgeon, meaning that they are able ‘… to conduct a comprehensive harm‒benefit analysis, including an assessment of whether the harm potentially arising from sedation of the donor animal is outweighed by the benefits’.^2^ The current requirements for a commercial feline blood bank (non‐food animal blood bank authorisation holder) in the UK require that blood donors are not sedated or anaesthetised for blood donation.^3^

Sedation of the feline blood donor is well described, and this is recognised as a standard method of enabling blood donation.^4^, ^5^ However, conscious feline blood donation has also been described in the literature.^6^ Preserving the welfare of blood donors is important, but universally recognised guidelines on how to do this are not available. Whether it is acceptable to sedate cats for blood donation, and if so, the optimum method of doing so, has not been determined.

Although there is a lack of clarity about the use of sedation in feline blood donation, it is recognised that if it is not used, this decreases the potential donor pool, as fewer cats are comfortable remaining still for the period of a donation.^4^, ^7^ The standard method of donation is using a needle in the jugular vein.^4^ This requires either heavy sedation or a compliant conscious/mildly sedated cat that is being gently restrained with the head raised. The primary aim of this study was to describe a novel method of blood donation using a standard short‐stay cephalic catheter that requires a lower level of restraint and minimal, or no, sedation. The secondary aim was to assess the success of this method when considering how frequently a complete donation was obtained, and also by assessing the free plasma haemoglobin level of the units of packed red blood cells obtained through this method when compared to those obtained via jugular venous donation.

## METHODS

This was a prospective observational study of all cats presenting as blood donors to a veterinary hospital between February and June 2023. To be registered as a blood donor, cats were assessed through history, physical examination, echocardiography, biochemical profile and complete blood count to be fit to donate. A feline leukaemia virus (FeLV) antigen and feline immunodeficiency virus antibody test was performed for each cat and polymerase chain reaction tests for *Bartonella* spp., *Mycoplasma haemofelis*, *Candidatus* Mycoplasma turicensis, *Candidatus* Mycoplasma haemominutum and FeLV provirus were also performed. Only cats that were negative for all diseases were enlisted as donors. The cats also underwent a behavioural assessment to determine if it was felt they were comfortable in a clinical room used exclusively for donation and if they appeared stressed or resented gentle handling, echocardiography and/or venepuncture (performed for the aforementioned tests), where samples were taken 45‒60 minutes after application of lidocaine/prilocaine cream (EMLA; Aspen). Any animals that were not tolerant or appeared stressed were not enrolled.

All cats presenting for blood donation had a short‐stay cephalic catheter placed. The skin over the cephalic vein was clipped, prilocaine/lidocaine cream (EMLA; Aspen) was applied and a non‐porous film was bandaged over this. A minimum of 45 minutes later, a 22‐gauge, 25 mm, fluorinated ethylene propylene catheter (short‐stay Jelco intravenous catheter) was placed. A pre‐flushed t‐connector was attached, and then the catheter was flushed. At this point, either a standard jugular venous blood donation (hereafter referred to as the jugular method) or collection of a blood donation via the cephalic catheter (hereafter referred to as the catheter method) was attempted. The cats where the jugular method was employed had prilocaine/lidocaine cream applied to the skin over the jugular vein a minimum of 45 minutes beforehand. The registered veterinary nurse carrying out the donation decided which method to use with the catheter method being chosen if it was suspected that jugular venous blood collection would not be tolerated.

Both the jugular and catheter methods used a sterilised closed collection system consisting of two 30 mL syringes, which contained citrate phosphate dextrose adenine anticoagulant and which were attached to a feline blood bag. For the catheter method, the collection system line was clamped, the butterfly needle was detached and then the collection system was attached directly to the t‐connector using a ‘no touch technique’ after alcohol application to the connection area. The catheter was aspirated and if blood flowed readily, then up to 53 mL of blood (a maximum of 12 mL/kg) was drawn. If the catheter method was used initially but further blood was then collected via the jugular method, the collection system line was clamped and a new sterile butterfly needle was attached using a no‐touch technique. Conversely, if the jugular method of collection was converted to the catheter method, the collection system line was clamped and the butterfly needle was removed, as per the standard catheter method noted above.

Whether the donation method was successful, the weight of the cat, the volume of blood obtained (mL/donor bodyweight kg and total mL) and any sedative drugs administered were recorded.

Once the donation was obtained, if the collection had been completed through one method alone, 1 mL of whole blood was retained within a syringe while the rest of the collected blood was transferred into the blood bag. This retained blood had total haemoglobin measured machine (HemoCue 201+). Samples were then centrifuged in microhaematocrit tubes and then plasma‐free haemoglobin was measured using a machine designed to measure low haemoglobin levels (HemoCue Plasma/Low Hb System). Percent haemolysis was calculated using the following equation:

$$\%\;\mathrm{Haemolysis}=\frac{\mathrm{Plasma}\;\mathrm{Hb}\;\left(g/\mathrm{dL}\right)\times \left(100-\;\mathrm{PCV}\right)}{\mathrm{Total}\;\mathrm{Hb}\;\left(g/\mathrm{dL}\right)}.$$

The blood volumes obtained (mL/kg and total mL) as well as haemolysis percentages were tested for normality using a Shapiro‒Wilk test. The data are presented as mean and standard deviation and total range if normally distributed and median and total range if not normally distributed. A *t*‐test was used to assess for a significant difference in percent haemolysis between packed red blood cell units collected via the catheter method and those collected via the jugular method.

## RESULTS

There were 46 feline donations performed over the study period.

In 12 cats, the catheter method was successfully used as an initial method for donation, and in one cat, the jugular method was attempted as an initial method for donation but was unsuccessful as no blood flow was obtained; thus, the catheter donation method was used. Cat weights ranged from 3.2 to 10.5 kg. Two of these 13 cats had 0.2 mg/kg butorphanol administered (one intravenously and one intramuscularly), one had 50 mg gabapentin administered orally prior to the journey to the hospital, and two cats had both drugs administered. Eight cats had no sedation drug administered. Mean blood donation volume was 9.0 mL/kg (standard deviation: 2.37 mL/kg, range: 4.9‒11.9 mL/kg), with a mean total unit volume of 45 mL (standard deviation: 9.74 mL, range: 29‒61 mL). The desired volume was obtained in eight (62%) cases and was not obtained in five (38%) cases.

In three cats, the catheter method was attempted as an initial method for donation unsuccessfully. In two cats, blood flow from the catheter was poor, and in another, the cat resented its feet being touched. In these three cases, a jugular donation was performed. One cat required sedation with 0.2 mg/kg intravenous butorphanol and the other two donated consciously. The cat requiring sedation did not complete a full donation, but in the other two cats, the desired donation volume was obtained.

In one cat, the catheter method was attempted as an initial donation method and was partially successful, with 40 mL obtained, but due to slow blood flow, the final 12 mL was obtained via jugular donation. This cat was administered 0.2 mL/kg butorphanol intramuscularly.

In 26 cats, the jugular method was successfully used as an initial method for donation. Cat weights ranged from 3.0 to 8.5 kg. One cat had 50 mg gabapentin administered orally prior to the journey to the hospital and one cat was administered 3 mg/kg ketamine, 0.2 mg/kg midazolam and 0.2 mg/kg butorphanol intravenously. Therefore, 24 cats donated consciously. Mean blood donation volume was 9.5 mL/kg (standard deviation: 1.89 mL/kg, range: 6‒12 mL/kg), with a median total unit volume of 51.6 mL (range: 30‒74 mL) obtained. The desired volume was obtained in 19 (73%) cases and was not obtained in seven cases (27%).

In three cats, the jugular method for donation was attempted as an initial method but was only partly successful, with only 7.5‒10 mL of blood being obtained; thus, the rest of the donation was from the intravenous catheter. In two cases, the desired volume was obtained (52 and 48 mL), and in one case, it was not (30 mL). Two cats were sedated with 0.2 mg/kg intramuscular butorphanol and one was sedated with 50 mg oral gabapentin.

Eleven of the donations were collected solely by the catheter method, and 11 that were collected solely by the jugular method had percent haemolysis of the collected blood calculated. The mean percent haemolysis for the catheter method was 0.21% (standard deviation: 0.11, range: 0.10%‒0.43%) and for the jugular method was 0.20% (standard deviation: 0.14, range: 0.01%‒0.54%), which were not significantly different.

## DISCUSSION

The primary aim of this study was to describe a technique for blood donation in cats via a standard cephalic catheter. The technique was shown to be effective, even in smaller cats. The benefit of this technique is that it allows donation with minimal restraint meaning that conscious or lightly sedated donation is more readily achievable. The need for conscious feline blood donation is great in the UK, given anaesthesia and sedation are prohibited for blood banks. Lack of sedation for donors may be beneficial in other scenarios, as it could be argued that donor welfare and morbidity would be improved as cats would not have to recover from drug administration. Agitation on recovery from sedation in feline blood donors using either a sevoflurane or a ketamine/midazolam/butorphanol protocol has been described.^8^ Interestingly, a recent study has reported significantly fewer adverse effects in blood donors sedated for donation than in those who were not sedated.^9^ The adverse events were primarily hypotensive and respiratory events in the hospital and lethargy or behavioural issues reported by the caregiver. The reason for the increased adverse events in the non‐sedated cats was unclear.^9^

In this study, although the majority of cats that donated via both methods were not sedated, mild sedation (gabapentin and butorphanol) was used fairly frequently, particularly in the catheter method group. The cats that donated blood via the catheter method were those felt less likely to tolerate a jugular donation; therefore, it would be anticipated that they would be more likely to require sedation than those thought likely to tolerate the jugular method. However, eight of the 13 cats donating via the catheter method did not require sedation, suggesting that this is a useful method for cats donating at blood banks, which may not tolerate standard jugular method donation. Also, the sedation used was relatively mild (butorphanol and/or gabapentin), allowing a likely quicker recovery and decreased morbidity compared to the use of heavier sedation (ketamine, midazolam and butorphanol or sevoflurane) where adverse donor effects have been described.^8^

For this method of blood collection to be accepted, the quality of the blood product obtained needs to be comparable to that obtained using the jugular method, both in terms of volume obtained and percent haemolysis. In the majority of cases, the desired volume was obtained via the catheter method, but this was not true for a sizable minority of cases. It is also worth noting that the method was attempted and needed to be abandoned in some cats due to lack of, or poor, blood flow. However, it was successfully used to ‘finish’ donations when the jugular method failed.

The aspiration of blood through a catheter could lead to increased risk of haemolysis. In human medicine, the use of an intravenous catheter has repeatedly been associated with an increased risk of blood sample haemolysis when compared to samples obtained via a butterfly needle.^10^, ^11^ Therefore, it was important to check haemolysis levels in the units obtained via the catheter donation method as elevated unit haemolysis can result in a haemolytic transfusion reaction.^12^ The percent haemolysis of the catheter method donations was well within the acceptable value for human packed red blood cells and that recommended for canine and feline cells at less than 1% (with the European human standard maximum for human units being 0.8%).^13^ Also, they were not significantly different from the values obtained for the jugular method donations. However, it should be noted that percent haemolysis of units does increase during storage of red cells.

This study has several limitations. The number of cats donating was fairly small and the percent haemolysis of units was not measured in all cases. Ideally, a randomised trial would have been performed to compare success rates in obtaining units and need for sedation. However, this study was performed in a hospital where blood donations were required for clinical practice, meaning that feline donor welfare was prioritised, so randomisation was not appropriate. Instead, the method used for donation was determined through assessment of the donor itself. Another limitation is that it is unclear how much the placement technique (including how proximal the catheter was placed) and individual cat factors (other than weight) affected likelihood of success for the catheter donation method, as these factors were not studied.

In conclusion, this study presents a novel method for obtaining blood donations in cats. Although not possible in all cats, intravenous catheter donation can be a successful method that may minimise stress to the feline donor and does not always require sedation. The quality of the blood product obtained appears to be acceptable.

## AUTHOR CONTRIBUTIONS

*Data collection, writing and reviewing of the manuscript*: Charlotte Russo. *Data assessment, writing and reviewing of the manuscript*: Karen Humm.

## CONFLICT OF INTEREST STATEMENT

The authors declare they have no conflicts of interest.

## FUNDING INFORMATION

This study was not funded.

## ETHICS STATEMENT

Ethical approval for this study was granted by the hospital's ethical review board (SR2024‐0047).

## Data Availability

The data that support the findings of this study are available from the corresponding author upon reasonable request.
